# New Naphthoquinone Terpenoids from Marine Actinobacterium, *Streptomyces* sp. CNQ-509

**DOI:** 10.3390/md16030090

**Published:** 2018-03-12

**Authors:** Jin-Soo Park, Hak Cheol Kwon

**Affiliations:** Natural Constituents Research Center, Korea Institute of Science and Technology (KIST), Gangneung, Gangwon-do 25451, Korea; jinsoopark@kist.re.kr

**Keywords:** marine Actinobacteria, natural product, hybrid terpenoid, meroterpenoid

## Abstract

A member of the marine streptomycete clade MAR4, *Streptomyces* sp. CNQ-509, has genetic potential for the biosynthesis of hybrid isoprenoids and produces several meroterpenoids such as naphterpin, nitropyrrolin and marinophenazine. Our research on the strain CNQ-509 led to the isolation of two new naphterpin derivatives (**1** and **2**) comprised of naphthoquinone and geranyl moieties along with the known terpenoid, debromomarinone. The two-dimensional structure of these compounds was determined through spectral data analysis using data from NMR, MS and UV spectroscopy. Furthermore, the full structures of **1** and **2** including absolute configurations were unequivocally established by a combination of NMR experiments and chemical modifications.

## 1. Introduction

**Figure 1:**
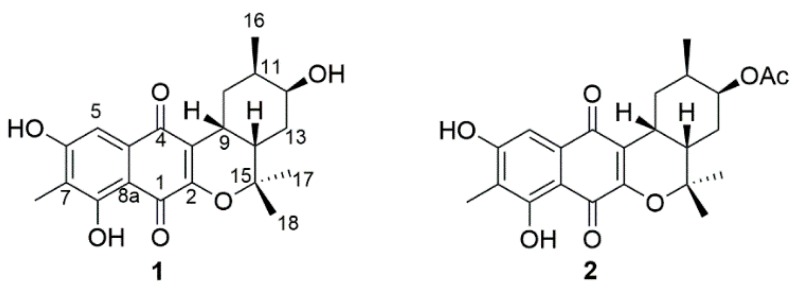
Structures of Compounds **1** and **2**.

A wide range of culture-independent approaches have revealed that Actinobacteria ubiquitously exist and form persistent populations in marine ecosystems [1]. These Actinobacteria have been continuously isolated from their marine environments and represent a prolific source for the discovery of interesting chemical scaffolds with biological activity in the last two decades (likewise, terrestrial Actinobacteria) [2]. Of the secondary metabolites that are derived from Actinobacteria, terpenoids are a large and diverse chemical class biosynthesized from the repetitive condensation of two simple five-carbon monomers: isopentenyl diphosphate and dimethylallyl diphosphate, each produced through two completely different pathways, the 2-*C*-methylerythritol 4-phosphate pathway and the mevalonate pathway, respectively [3]. As a distinct marine actinobacterial lineage within the genus *Streptomyces*, the streptomycete clade MAR4 is excellently proficient at producing diverse hybrid terpenoid skeletons such as polyketide-terpenoid molecules [4]. The strain CNQ-509, isolated from the marine sediment collected off La Jolla, California, also belongs to this group [4]. Since our previous report on unusual farnesyl-α-nitropyrroles (nitropyrrolins) [5], further chemical investigation of the strain CNQ-509 had led to the discovery of two new naphterpin derivatives (**1** and **2**), both naphthoquinone-based meroterpenoids (Figure 1). Herein, we report the structure elucidation of the isolated compounds including their absolute stereochemistry and radical scavenging activities.

## 2. Results and Discussion

**Figure 2:**
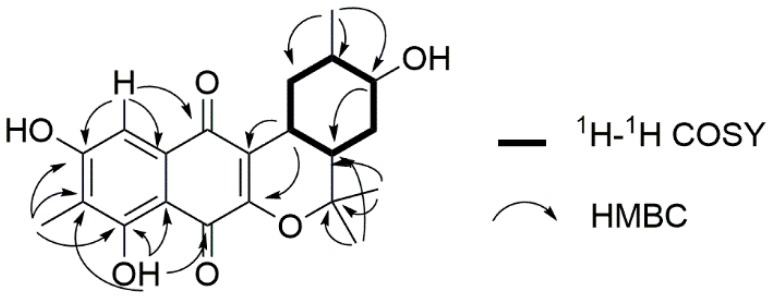
^1^H-^1^H COSY (bold lines) and HMBC correlation (arrows) for the construction of the planar structure of Compound **1.**

Compound **1** was obtained as a yellow amorphous solid. The molecular formula was determined as C_21_H_24_O_6_ on the basis of the pseudomolecular ion peak at *m*/*z* 371.1497 [M − H]^−^ in the high-resolution fast atom bombardment mass spectrometry (HR-FAB-MS) spectrum indicating ten degrees of unsaturation. Our in-house UV database implied that Compound **1** had a naphthoquinone-like chromophore based on its long wavelength absorption at 399 nm (ε 1740), which is a value similar to that of the UV characteristics of naphthoquinone compounds [6,7]. The direct connectivity between protons and carbons was determined by combining HSQC spectra data with ^1^H and ^13^C-NMR data (Table 1), to discriminate exchangeable protons. In the ^1^H-NMR spectrum, the naphthoquinone moiety was deduced from the presence of four singlet proton signals (δ_H_ 2.18, 7.20, 7.63, 12.23) representative of an aromatic methyl, an aromatic methine and two phenolic hydroxyl protons indicating the substitution of two hydroxyl groups and one methyl group. The naphthoquinone part was assured through further analysis of ^1^H-^13^C couplings observed in the HMBC spectrum as shown in Figure 2. Apart from naphthoquinone, the 1-hydroxy-2-methyl cyclohexane ring moiety was easily identified through the presence of successive ^1^H-^1^H COSY cross peaks from H_3_-16 (δ_H_ 0.90) to H_2_-10 (δ_H_ 2.67, 1.78) extending to a monoterpenoid side chain, connected with the naphthoquinone moiety. This identification was supported by HMBC correlations from H_3_-17 (δ_H_ 1.49)/H_3_-18 (δ_H_ 1.33) to C-14 (δ_C_ 35.5)/C-15 (δ_H_ 81.2)) and H-9 (δ_H_ 3.19) to C-2 (δ_C_ 156.0)/C-3 (δ_C_ 121.2) (Figure 2). Thus, the planar structure of **1** was determined to be a naphterpin derivative, 10-dihydro-12-hydroxynaphterpin, as shown in Figure 1.

**Figure 3:**
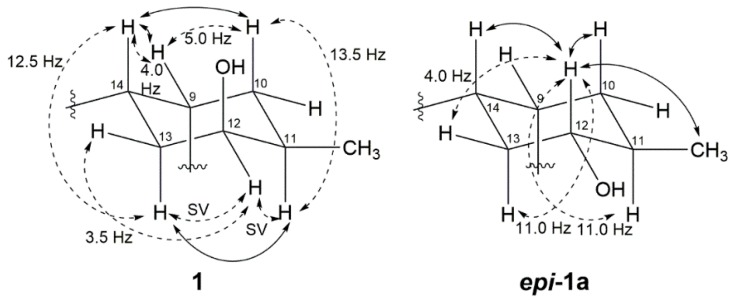
Relative stereochemistry of C-9 to C-14 of Compound **1** and ***epi***-**1a**. Important NOEs are illustrated with solid arrows and vicinal couplings with dashed arrows. Coupling constants are given in hertz (SV: small value).

The vicinal coupling constant and NOE correlation allowed the determination of the relative configuration of the cyclohexane ring in **1**. Large ^3^*J*_H,H_ between H-10b (δ_H_ 1.78)/H-11 (*J* = 13.5 Hz) and H-14/H-13a (δ_H_ 2.01) (*J* = 12.5 Hz) suggests that these protons are in a diaxial orientation, while the small coupling constants of H-10a/H-9, H-9/H-14, H-14/H-13b and H-13a/H-12 imply that H-9, H-10a, 12-H and H-13b are in equatorial positions. Thus, a chair form cyclohexane ring was established as shown in Figure 3. Further evidence for this structure is given by the NOE correlation between H-10b/H-14 and H-11/H-13b.

Compound **2** was also isolated as a yellow amorphous solid, and the molecular formula was deduced to be C_23_H_26_O_7_ through HR-FAB-MS analysis (obsd. [M − H]^−^ at *m*/*z* 413.1602, calcd. [M − H]^−^ 413.1600) in combination with ^1^H and ^13^C-NMR data (Table 1). The ^1^H-NMR of **2** was very similar to that of **1**, except for a methyl singlet signal at 2.10 ppm indicating the presence of an acetyl group. The difference in molecular formula, C_2_H_2_O, also strengthened the conclusion that **2** was acetylated from **1**. The substituted position of the acetyl functional group was determined by ^1^H-^13^C long-range couplings from a singlet methyl proton and the oxymethine proton H-12 (δ_H_ 5.00) to an ester carbonyl carbon (δ_C_ 170.8). Therefore, the planar structure of **2** was established to be acetylated at C-12 of **1** as shown in Figure 1. 

**Figure 4:**
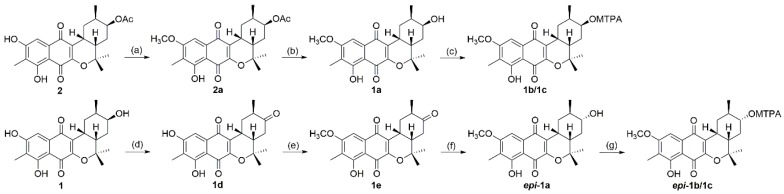
Scheme of the stereochemistry of **1** and **2**. (**a**,**e**) methyl iodide (MeI), K_2_CO_3_, acetone; (**b**) K_2_CO_3_, methanol; (**c**,**g**) (*R*)/(*S*)-α-methoxy-α-(trifluoromethyl)phenylacetyl (MTPA)-Cl, DMAP, pyridine-*d*_5_; (**d**) pyridinium chlorochromate (PCC), CH_2_Cl_2_; (**f**) NaBH_4_, methanol.

**Figure 5:**
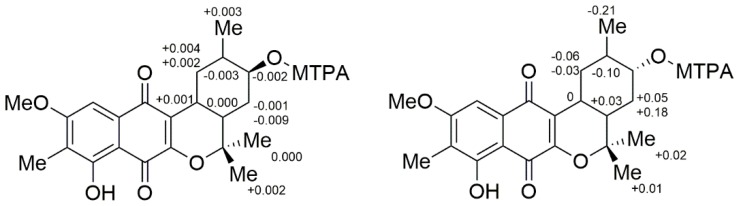
Δδ_S-R_ values for the Mosher esters **1b**/**1c** (left) and ***epi*-1b**/**1c** (right).

To solve the absolute stereochemistry of the structures **1** and **2**, α-methoxy-α-(trifluoromethyl)phenylacetyl (MTPA) ester was introduced to deacetylated **2** (**1a**), prior to protection through methylation at 6-OH, as shown in Figure 4. Contrary to our expectations, the Δδ*_S_*_-*R*_^1^H-NMR values for the Mosher esters **1b**/**1c** were irregularly distributed; thus, the absolute configuration of C-12 was not determined. This inconstant deviation might occur due to the axial alcohol orientation of C-12 as previously reported [8]. The limitations of the modified Mosher’s method in the case of the axial alcohol were avoided by replacing the equatorial hydroxyl group (***epi*-1a**) using pyridinium chlorochromate (PCC) oxidation, followed by the reduction of **1** with NaBH_4_ (Figure 4) [9]. The equatorial orientation of 12-H in ***epi*-1a** was assured by the large ^3^*J*_H-H_ values of H-12/H-11 (11.0 Hz) and H-12/H-13a (11.0 Hz), as well as NOE correlations between H-12 and H-10b/H-14, not observed in the NOESY spectrum of **1a** (Figure 3). Subsequently, the absolute configuration of C-12 in ***epi*-1a** was assigned as *R* based on a modified Mosher analysis, which showed a diagnostic ^1^H-NMR chemical shift difference (Δδ*_S-R_*) between MTPA esters of ***epi*-1a** (Figure 5). Therefore, C-12 in **1** was assigned as having an *S* configuration. Based on relative stereochemistry, the absolute configurations for all of the asymmetric centers in **1** and **2** were thus assigned as *9R*, *11R*, *12S* and *14S*, and the entire structure was fully determined.

1,4-Naphthoquinone scaffolds are often found in nature and have antioxidant properties, typically through vitamin K [10,11]. The close structure of **1** and **2**, naphterpin, was previously reported as having potent antioxidant properties, inhibiting lipid peroxidation in rat live microsomes [12]. Thus, we supposed that the isolated compounds might also have antioxidant properties. To evaluate the free radical scavenging activities of **1** and **2**, we tested the on-line ABTS^+^ assay with an HPLC system coupled with an additional pump supplying radical reagents, to rapidly determine any antioxidant effects. From the combined UV (positive signals) and ABTS^+^ quenching (negative signals) chromatograms, both compounds showed free radical scavenging activity (Figure S13).

Compounds **1** and **2** are naphthoquinone-based meroterpenoids. Other compounds in this group include naphterins [12], marinones [6], napyradiomycins [13], merochlorins [14] and naphthablins [15] isolated from actinomycetes. Since the discovery of the aromatic substrate prenyltransferase NphB (former Orf2) in the naphterpin biosynthetic gene cluster, it has been widely accepted that Actinobacteria-derived meroterpenoids are produced through a putative biosynthetic pathway, in which the polyketide-derived aromatic core is substituted with geranyl pyrophosphate or farnesyl pyrophosphate and subsequently modified by cyclization, hydroxylation and methylation [16]. Compared to the detailed studies on terpene biosynthesis in these meroterpenoids, the polyketide acceptor used to bind isoprenyl group has not been completely elucidated [17,18,19]. Recently, a unifying paradigm for naphthoquine meroterpenoid was suggested and demonstrated: biosynthesis occurs through the prenylation of tetrahydroxynaphthalene and α-hydroxy ketone rearrangement catalyzed by vanadium-dependent haloperoxidase (VHPO) [20]. As VHPO homologs were found in the biosynthetic gene cluster of the genome of the strain CNQ-509, Compounds **1** and **2** might also be biosynthesized via the same pathway of napyradiomycin and merochlorin.

A member of MAR4, the strain CNQ-509 produces diverse terpenoid structures connected with a (hetero)aromatic core (pyrrole, phenazine and naphthoquinone) [5,21]. Furthermore, genome analysis also led us to predict that this strain has the genetic potential for synthesizing terpenoid compounds via several biosynthetic gene clusters containing prenyltransferase-encoding genes [21,22,23]. This suggests that marine Actinobacteria, especially MAR4, are remarkable biological resources for terpenoid-related chemical diversity (terpenome) [24].

## 3. Materials and Methods

### 3.1. General Experimental Procedures

Optical rotations and the UV spectrum were measured on a Model 343 polarimeter (PerkinElmer, Waltham, MA, USA) and a Lambda 35 UV/vis spectrophotometer (PerkinElmer, Waltham, MA, USA), respectively. ^1^H, 13C and 2D NMR spectral data were obtained in CDCl_3_ on a VNMRS 500 NMR spectrometer (Agilent Technologies, Santa Clara, CA, USA). Low-resolution ESI-MS were measured on an Agilent 1100 LC/MS system (Agilent Technologies, Santa Clara, CA, USA) with a Luna C18(2) 5-μm column (4.6 mm × 150 mm, flow rate 0.7 mL/min) (Phenomenex, Torrance, CA, USA). High-resolution mass spectral data were acquired on a JMS-AX505WA mass spectrometer (JEOL Ltd., Akishima-shi, Tokyo, Japan). A Lichroprep RP-18 (Merck, Darmstadt, Germany) was used for the flash column chromatography. Semipreparative HPLC separations were performed using a 321 HPLC system (Gilson Inc., Middleton, WI, USA) with a Luna C18(2) 10-μm column (10 × 250 mm) at a flow rate of 4 mL/min. A 1525 HPLC-PDA system (Waters, Milford, MA, USA) with a Luna C18(2) 5-μm column (4.6 mm × 150 mm) was used for the routine analysis of extracts and fractions. HPLC-grade solvents were used for all chromatographic analyses.

### 3.2. Cultivation and Extraction of Strain CNQ-509

Strain CNQ-509, isolated from a marine sediment sample (La Jolla, CA, USA), was grown on marine agar 2216 (Difco, Detroit, MI, USA) for 7 days at 27 °C and cultured in 200 mL of A1 medium (10 g of starch, 4 g of peptone, 2 g of yeast extract in 1 L of seawater) in a 500-mL Erlenmeyer flask for 3 days at 27 °C with shaking at 200 rpm. For the production of secondary metabolites, the 200 mL of culture broth of the strain were transferred to a 7-L fermenter (LiFlus GR, BioTron Inc., Bucheon, Korea) containing 4 L of A1 medium. The cultivation was performed for 7 days at 27 °C while stirring at 400 rpm under a 5-L/min aeration rate. The secondary metabolites produced by the strain were analyzed by HPLC-MS at various time points over the course of the 7 days. After culturing in six replicate fermenters, the culture extract (2.8 g) was obtained by adding Amberlite XAD-7 adsorbent resin (20 g/L) and eluting with methanol.

### 3.3. Isolation and Purification of Naphterpins D and E (***1***, ***2***)

The extract (2.8 g) was fractionated by C18 flash column chromatography using methanol-water solvent mixtures (10%, 30%, 50%, 70%, 90% and 100% methanol in water, each 400 mL) as mobile phases. The 70% and 90% methanol fractions contained Compound **1** and **2**, respectively, and were purified by prep-HPLC with a Luna C18 (2) column using an acetonitrile-water isocratic condition (flow rate 10 mL/min) to yield Compounds **1** and **2**. Additionally, debromomarinone was isolated from a 100% methanol fraction.

Compound **1**: yellow amorphous solid; [α]${}_{D}^{24}$ −120 (*c* 0.05 MeOH); UV (λ_max_, MeOH) (log ε) 215 nm (4.35), 268 nm (4.03), 314 nm (3.83), 399 nm (3.24); HR-FAB-MS *m*/*z* 371.1497 [M − H]^−^ (calcd. for C_21_H_23_O_6,_ 371.1495); ^1^H and ^13^C-NMR spectroscopic data; see Table 1.

Compound **2**: yellow amorphous solid; [α]${}_{D}^{24}$ −115 (*c* 0.04 MeOH); UV (λ_max_, MeOH) (log ε) 215 nm (4.31), 268 nm (4.02), 317 nm (3.80), 400 nm (3.20); HR-FAB-MS *m*/*z* 413.1602 [M − H]^−^ (calcd. for C_23_H_25_O_7,_ 413.1600); ^1^H and ^13^C-NMR spectroscopic data; see Table 1.

### 3.4. Methylation of ***2** (**2a**)* and Deacetylation of ***2a** (**1a**)*

To a solution of **2** (5 mg) in acetone, MeI (50 μL) and K_2_CO_3_ (s) (50 mg) were added, and the mixture was stirred for 18 h. After a standard aqueous workup, the product was subjected to preparative reversed HPLC to yield Compound **2a** (6 mg), confirmed by ESI-MS *m*/*z* 429.1 [M + H]^+^. Subsequently, **2a** was dissolved in methanol with K_2_CO_3_. The mixture solution was heated to obtain deacetylated **2a** (**1a**, 3 mg).

**1a**: yellow solid; ESI-MS *m*/*z* 387.1 [M + H]^+^; ^1^H-NMR: δ 12.10 (1H, s, 8-OH), 7.19 (1H, s, H-5), 3.99 (3H, s, OCH_3_), 3.83 (1H, m, H-12), 3.19 (1H, m, H-9), 2.72 (1H, m, H-10a), 2.15 (3H, s, 7-CH_3_), 1.98–2.09 (2H, m, H-14, H-13a), 1.75–1.85 (1H, m, H-10b), 1.52 (1H, s, 17-CH_3_), 1.33–1.42 (2H, m, H-11, H-13b), 1.33 (3H, s, 18-CH_3_), 0.92 (3H, d, 16-CH_3_).

### 3.5. Oxidation of ***1** (**1d**)* and Methylation of ***1d** (**1e**)*

To solution of **1** (3 mg) in CH_2_Cl_2_, pyridinium chlorochromate (PCC, 3 mg) was added. This mixture was stirred for 12 h at room temperature. After the usual workup, the ketone was isolated by reverse HPLC to create Compound **1d** (2.5 mg): ESI-MS *m*/*z* 371.2 [M + H]^+^.

Methylation of **1d** was prepared by the aforementioned method for **2a** to yield **1e** (2.0 mg): ESIMS *m*/*z* 383.1 [M − H]^−^.

### 3.6. Reduction of Compound ***1e*** *(***epi*****-1a**)*

Two milligrams of **1e** were transferred to a vial containing 1 mg of NaBH_4_ and 1 mL of MeOH, and the reaction mixture was stirred for 1 h. After the usual workup, the reduced product was obtained by reverse HPLC and identified via NOE experiments to be an epimer of **1a** (***epi*-1a**): ESI-MS *m*/*z* 387.1 [M + H]^+^; ^1^H-NMR: δ 12.09 (1H, s, 8-OH), 7.18 (1H, s, H-5), 3.99 (3H, s, 6-OCH_3_), 3.18 (1H, ddd, H-9), 3.10 (1H, m, H-12), 3.05 (1H, dt, H-10a), 2.15 (3H, s, 7-CH_3_), 2.11 (1H, dq, H-14), 1.83 (1H, ddd, H-13a), 1.55 (3H, s, H-17), 1.32 (3H, s, H-18), 1.26 (1H, ddd, H-11), 1.06–1.15 (2H, m, H-10b and H-13b), 0.99 (3H, d, H-16).

### 3.7. Preparation of MTPA Esters

Compound **1a** *and **epi*****-1a** were divided into two portions, and each was dissolved in 600 μL of pyridine-*d*_5_ in a 5-mm NMR tube. A slight excess of dimethylaminopyridine was added to each NMR tube. The samples were then treated with 5 μL of (*R*)-α-methoxy-α-(trifluoromethyl)phenylacetyl chloride (MTPA-Cl) and 5 μL of (*S*)-MTPA-Cl at room temperature. After 12 h, the reaction was completed, and ^1^H-NMR and COSY spectra for (*S*)-Mosher esters (**1b** and ***epi*-1b**) and (*R*)-Mosher esters (**1c** and ***epi*-1c**) were recorded.

**1b**: ^1^H-NMR (500 MHz, pyridine-*d*_5_): δ 3.376 (1H, H-9), 3.250 (1H, H-10a), 1.838 (1H, H-10b), 1.729 (1H, H-11), 5.397 (1H, H-12). 2.188 (1H, H-13a), 1.537 (1H, H-13b), 2.042 (1H, H-14), 0.891 (3H, H-16), 1.272 (3H, H-17), 1.329 (3H, H-18).

**1c**: ^1^H-NMR (500 MHz, pyridine-*d*_5_): δ 3.375 (1H, H-9), 3.246 (1H, H-10a), 1.836 (1H, H-10b), 1.732 (1H, H-11), 5.399 (1H, H-12), 2.189 (1H, H-13a), 1.546 (1H, H-13b), 2.042 (1H, H-14), 0.888 (3H, H-16), 1.272 (3H, H-17), 1.327 (3H, H-18).

***epi*-1b**: ^1^H-NMR (500 MHz, pyridine-*d*_5_): δ 3.22 (1H, H-9), 3.44 (1H, H-10a), 1.44 (1H, H-10b), 1.61 (1H, H-11), 4.98 (1H, H-12), 2.44 (1H, H-13a), 1.55 (1H, H-13b), 1.89 (1H, H-14), 0.80 (3H, H-16), 1.26 (3H, H-17), 1.40 (3H, H-18).

***epi*-1c**: ^1^H-NMR (500 MHz, pyridine-*d*_5_): δ 3.22 (1H, H-9), 3.50 (1H, H-10a), 1.47 (1H, H-10b), 1.71 (1H, H-11), 4.98 (1H, H-12), 2.39 (1H, H-13a), 1.37 (1H, H-13b), 1.86 (1H, H-14), 1.01 (3H, H-16), 1.24 (3H, H-17), 1.39 (3H, H-18).

### 3.8. On-Line Detection of Radical Scavenging Activity

The radical scavenging activity of Compounds **1** and **2** was determined using the on-line ABTS^+^ decolorization assay according to the previously reported method, using a preparation of 2 mM ABTS^+^ stock solution containing 3.5 mM potassium persulfate, incubated overnight in the dark at room temperature [25]. Briefly, 10 μL of two compounds (0.5 mM) were injected into the online HPLC-ABTS system using a Luna C18(2) 5 μm column and a solvent gradient of 10–100% acetonitrile over 20 min (flow rate 1.0 mL/min) combined with a supplement of the ABTS radical solution (flow rate 0.5 mL/min). The chromatogram was measured at 215 nm and 268 nm as a positive signal, and the decrease of ABTS radical was recorded at 734 nm as a negative signal. Trolox (0.5 mM) was used as the positive control. 

## 4. Conclusions

In summary, a chemical investigation was carried out on the marine Actinobacterium strain CNQ-509, which resulted in the isolation of two naphthoquinone-based terpenoids (Compounds **1** and **2**) along with the previously reported debromomarinone. The planar structures of **1** and **2** were determined. Furthermore, their absolute chemical structures were unambiguously established through NOESY experiments and chiral derivatization achieved through the introduction of an equatorial hydroxyl group. Similar to other naphthoquinone molecules, **1** and **2** also exhibited radical scavenging activities. In this context, our report endorses marine Actinobacteria, especially MAR4, as a prolific source of hybrid isoprenoid natural products.

## Figures and Tables

**Table 1:** ^1^H and ^13^C-NMR data of **1** and **2** (500 MHz, CDCl_3_).

Position	Compound 1		Compound 2	
	δ_H_, mult (*J* in Hz)	δ_C_	δ_H_, mult (*J* in Hz)	δ_C_
1		182.5		182.6
2		156.0		155.6
3		121.2		121.1
4		184.6		183.9
4a		131.7		131.9
5	7.20 s	108.1	7.06 s	107.6
6		161.3		160.5
7		117.0		116.7
8		162.5		162.3
8a		108.0		108.5
9	3.19 m	30.5	3.21 m	30.3
10	2.67 ddd (14.0, 2.5, 2.5) 1.78 ddd (13.5, 13.5, 5.0)	27.5	2.79 dt (14.0, 2.5) 1.74 ddd (13.5, 13.5, 4.0)	28.6
11	1.37 m	31.4	1.43 m	30.4
12	3.84 m	69.8	5.00 ddd(2.0, 2.0, 2.0)	72.2
13	2.01 dt (12.5, 3.5) 1.35 m	30.4	2.04 m 1.34 m	28.0
14	2.06 dt (12.5, 4.0)	35.5	1.91 ddd(13.0, 5.0, 4.5)	36.3
15		81.2		80.8
16	0.90 d (7.0)	17.9	0.83 d (6.5)	17.7
17	1.49 s	25.7	1.49 s	25.8
18	1.33 s	25.3	1.33 s	25.2
7-CH_3_	2.18 s	7.8	2.18 s	7.7
6-OH	7.63 s		6.02 s	
8-OH	12.23 s		12.3 s	
12-OH	2.02 s			
OAc			2.10 s	21.3
-COO-				170.8

## Supplementary Materials

The following are available online at http://www.mdpi.com/1660-3397/16/3/90/s1: Figures S1–S6: ^1^H, ^13^C, COSY, HSQC, HMBC and NOESY NMR data of **1** in CDCl_3_; Figures S7 and S8: ^1^H and ^13^C-NMR data of **2** in CDCl_3_; Figures S9–S12: 1H-NMR data of **1a**, ***epi*-1a** in CDCl_3_, ***epi*-1b** and ***epi*-1c** in pyridine-*d*_5_; Figure S13: On-line ABTS^+^ assay of Trolox, **1** and **2**.
