# Supplementary material for: New Naphthoquinone Terpenoids from Marine Actinobacterium, Streptomyces sp. CNQ-509
Source: Mar Drugs. 2018 Mar 12;16(3):90. doi: 10.3390/md16030090 (PMC5867634; doi:10.3390/md16030090)

Supplementary Material:

New Naphthoquinone Terpenoids from Marine Actinomycete, *Streptomyces* sp. CNQ-509

Jin-Soo Park and Hak Cheol Kwon *

Natural Constituents Research Center, Korea Institute of Science and Technology (KIST), Gangneung, Gangwon-do 25451, Republic of Korea; jinsoopark@kist.re.kr(J.-S.P.); hkwon@kist.re.kr(H.C.K.);

* Correspondence: hkwon@kist.re.kr; Tel.: +82-33-650-3504

Table of Contents

Figure S1. ^{1}H spectrum of **1** in $CDCl_3$

Figure S2. ^{13}C NMR spectrum of **1** in $CDCl_3$

Figure S3. COSY spectrum of **1** in $CDCl_3$

Figure S4. HSQC NMR spectrum of **1** in $CDCl_3$

Figure S5. HSQC NMR spectrum of **1** in $CDCl_3$

Figure S6. NOESY NMR spectrum of **1** in $CDCl_3$

Figure S7. ^{1}H spectrum of **2** in $CDCl_3$

Figure S8. ^{13}C NMR spectrum of **2** in $CDCl_3$

Figure S9. ^{1}H spectrum of **1a** in $CDCl_3$

Figure S10. ^{1}H spectrum of ***epi*-1a** in $CDCl_3$

Figure S11. ^{1}H spectrum of ***epi*-1b** in pyridine-d_5

Figure S12. ^{1}H spectrum of ***epi*-1c** in pyridine-d_5

Figure S13. On-line-$ABTS^{+}$ assay of Trolox (a), **1** (b) and **2** (c).

Figure S1. ^{1}H spectrum of **1** in $CDCl_3$

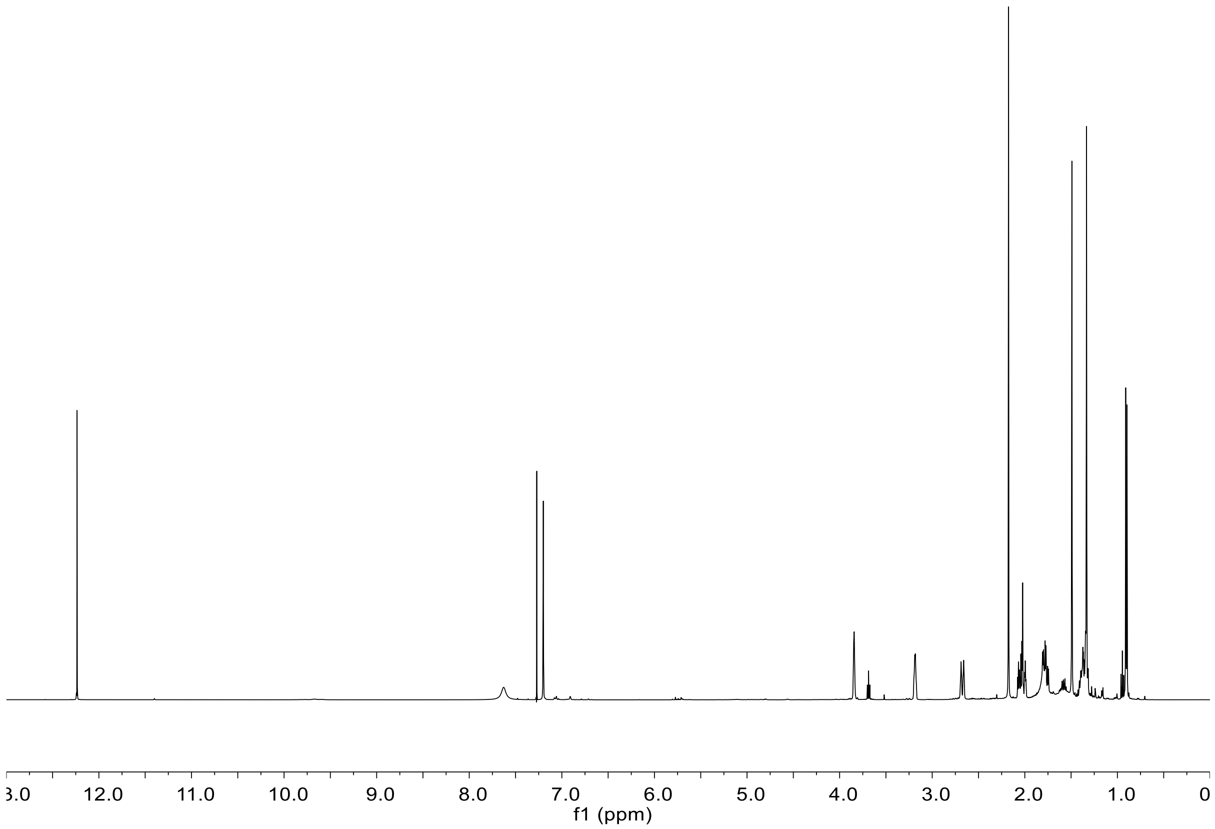

Figure S2. ^{13}C NMR spectrum of **1** in $CDCl_3$

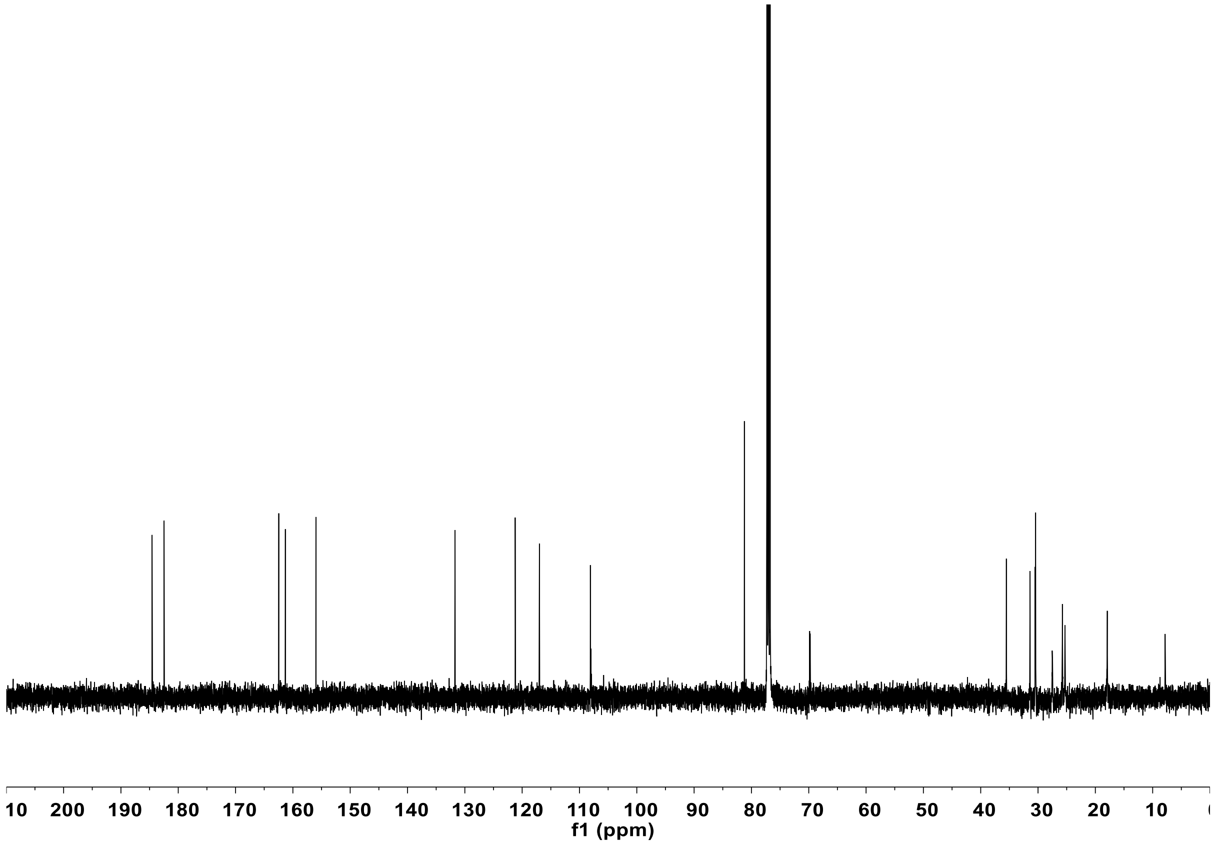

Figure S3. COSY spectrum of **1** in $CDCl_3$

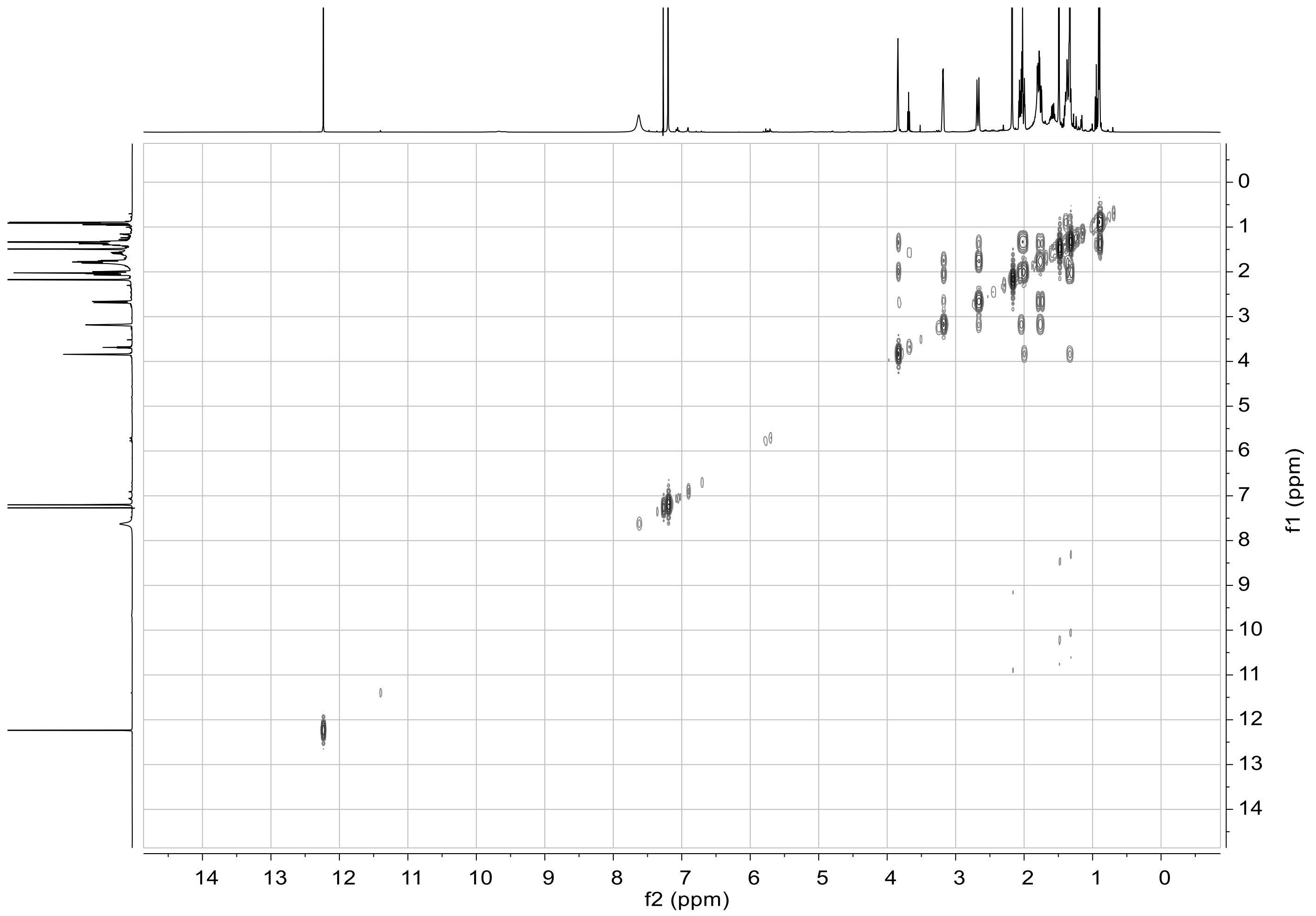

Figure S4. HSQC NMR spectrum of **1** in $CDCl_3$

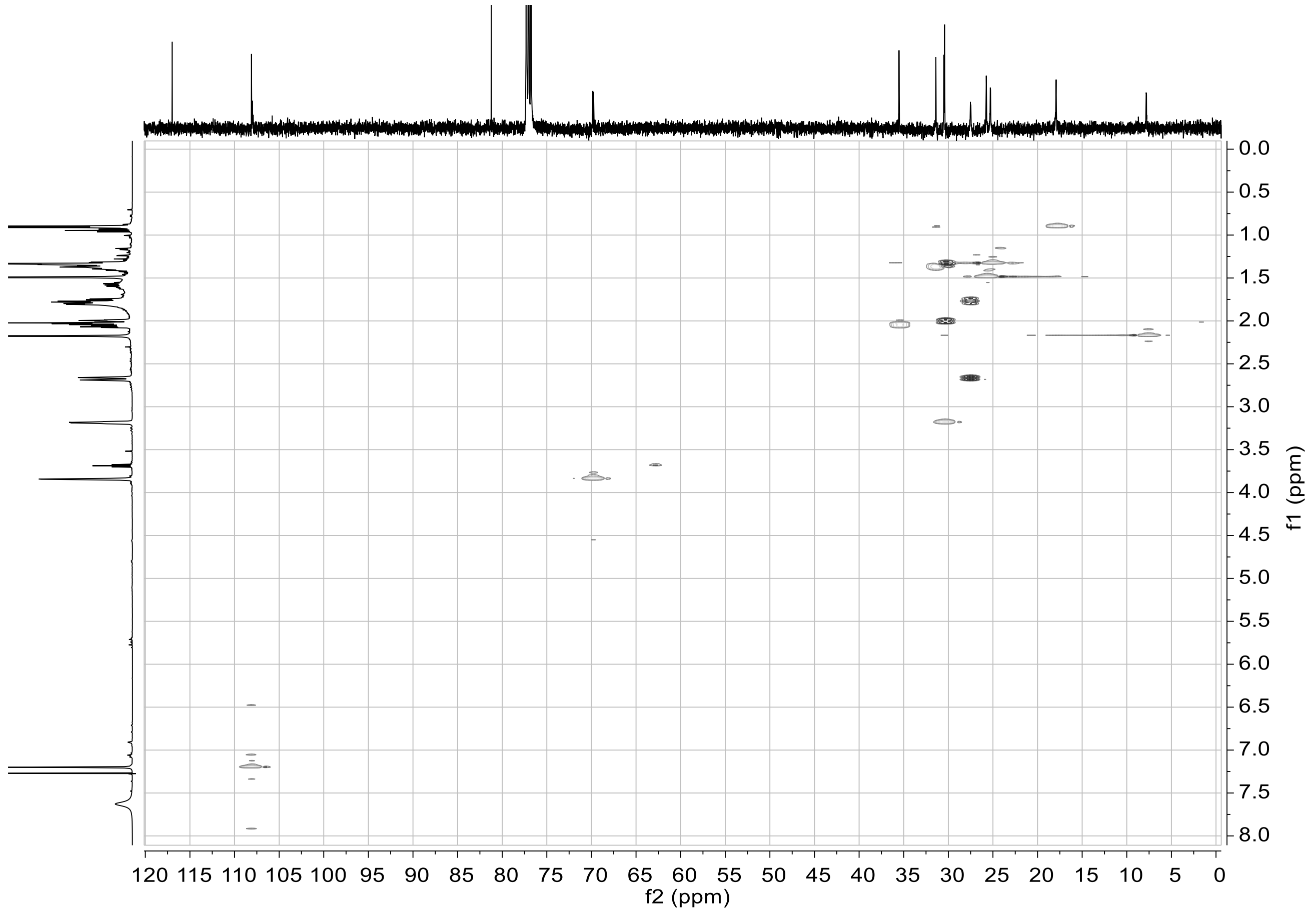

Figure S5. HSQC NMR spectrum of **1** in $CDCl_3$

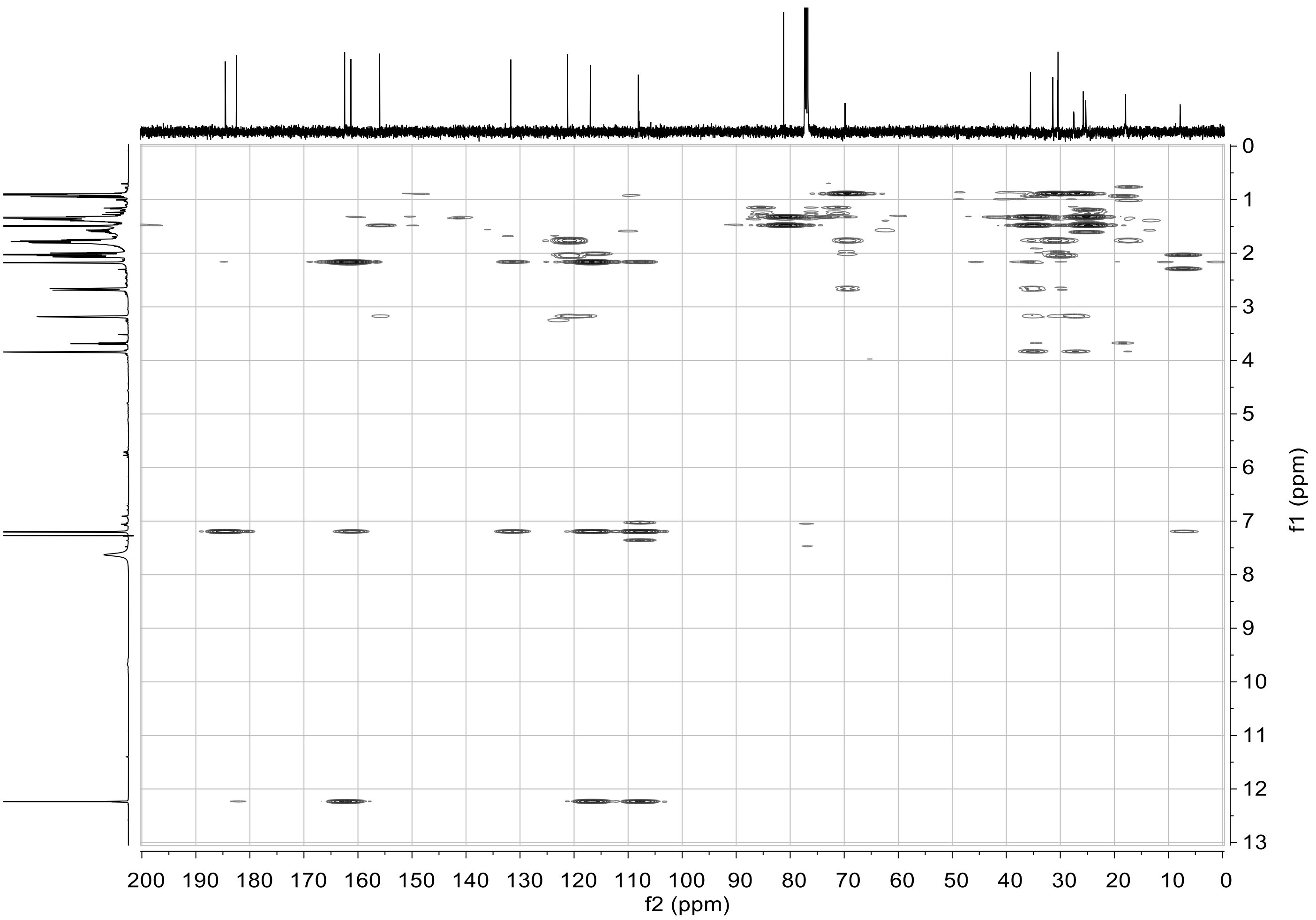

Figure S6. NOESY NMR spectrum of **1** in $CDCl_3$

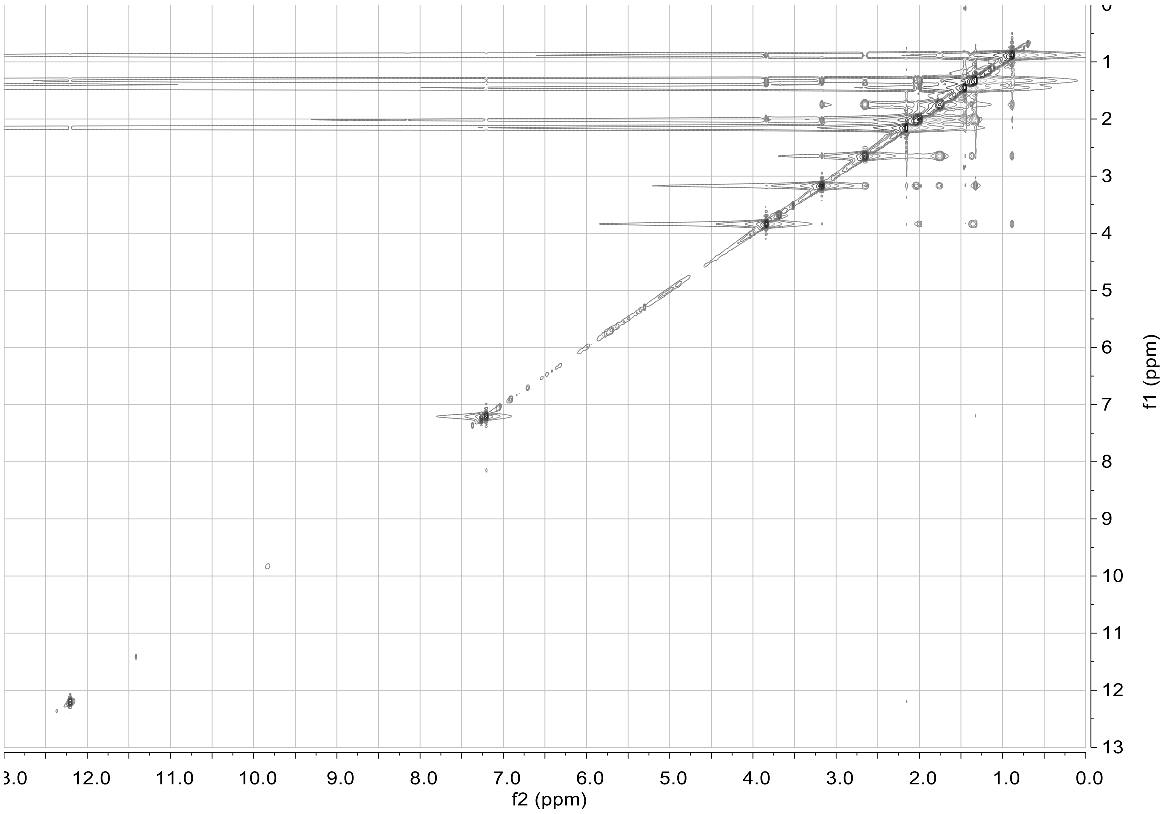

Figure S7. ^{1}H spectrum of **2** in $CDCl_3$

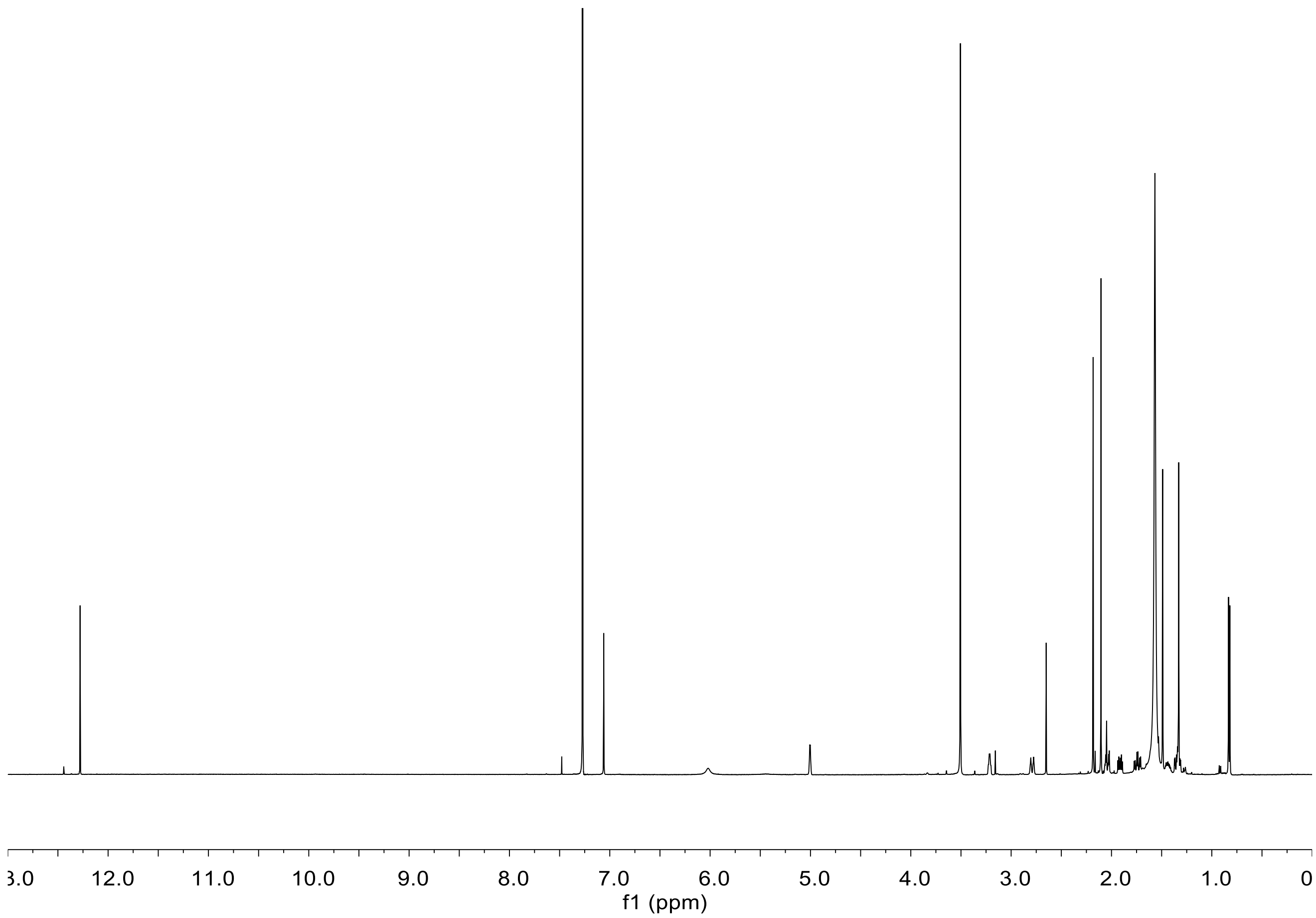

Figure S8. ^{13}C NMR spectrum of **2** in $CDCl_3$

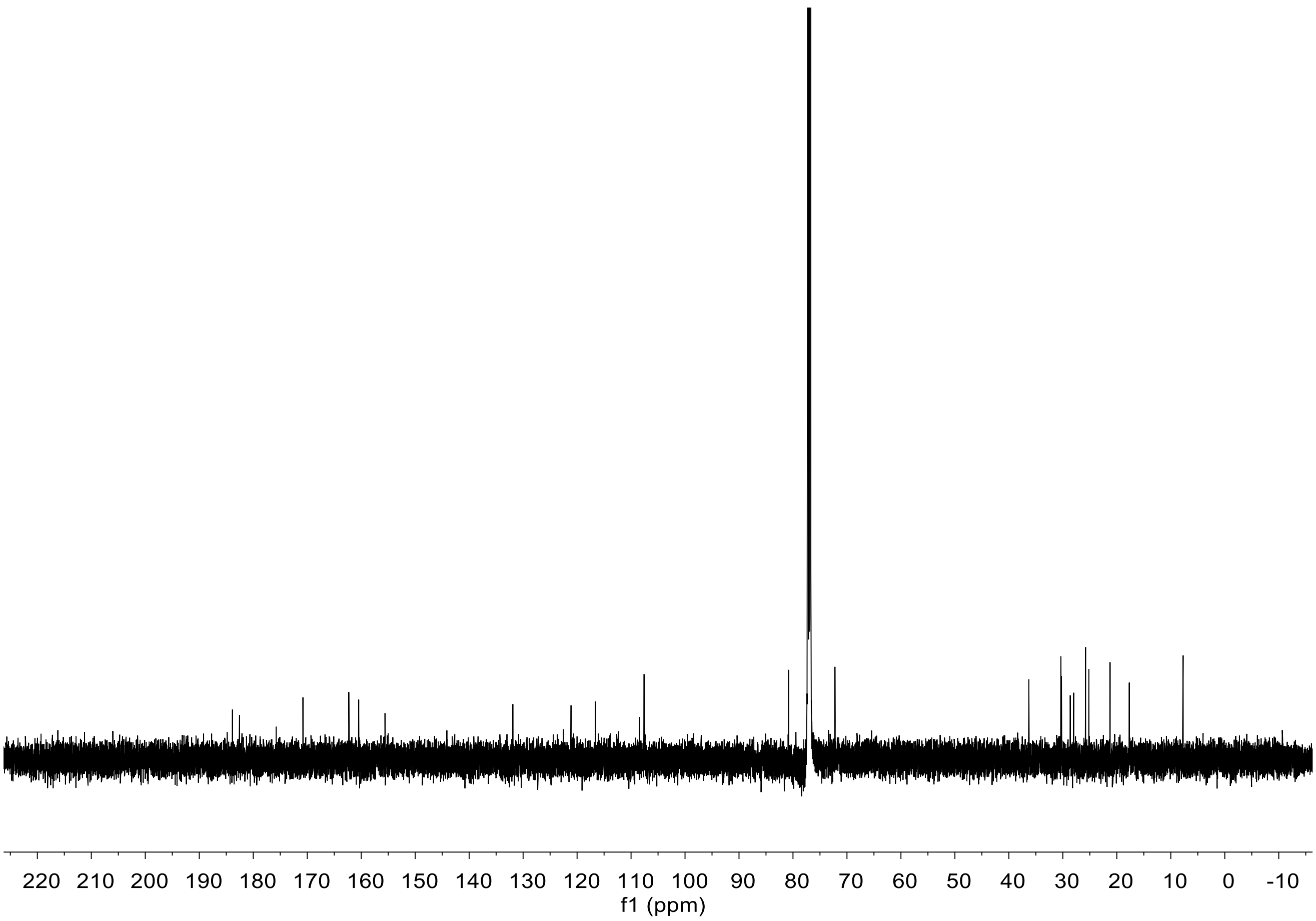

Figure S9. ^1H spectrum of **1a** in $CDCl_3$

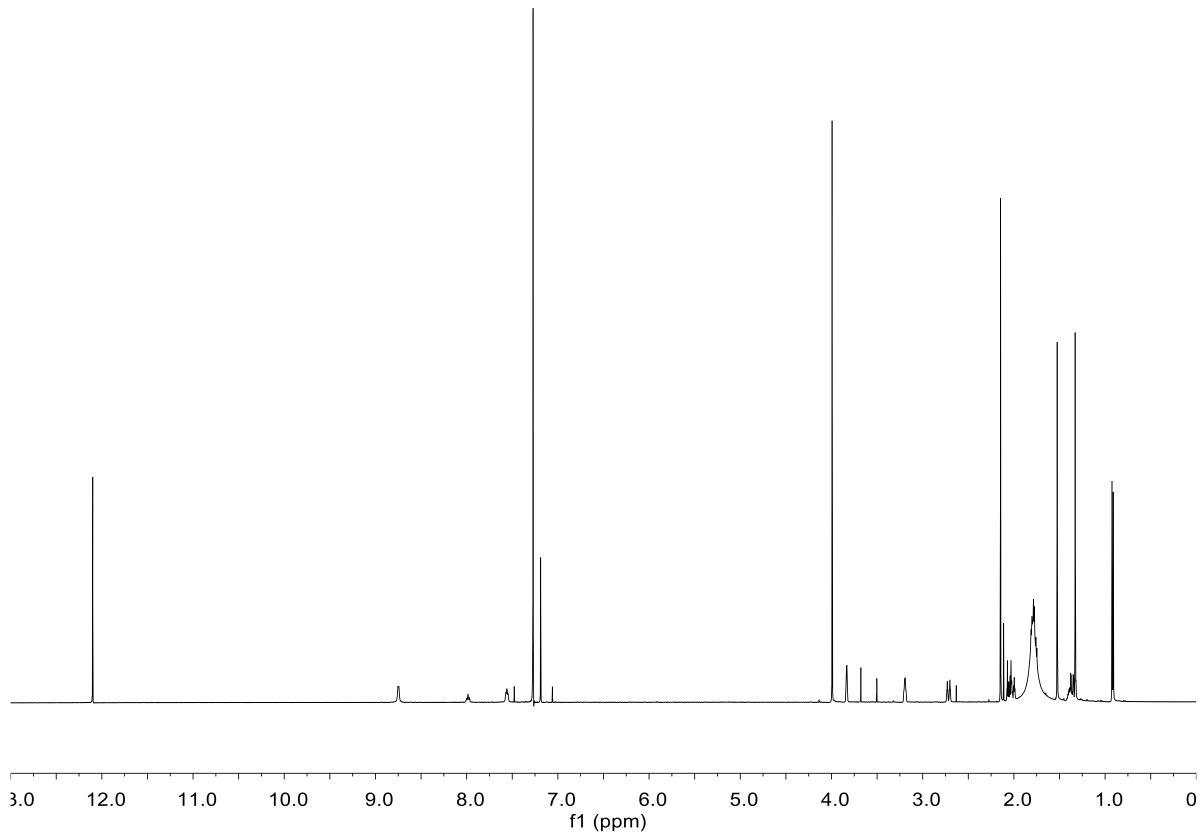

Figure S10. ^1H spectrum of ***epi*-1a** in $CDCl_3$

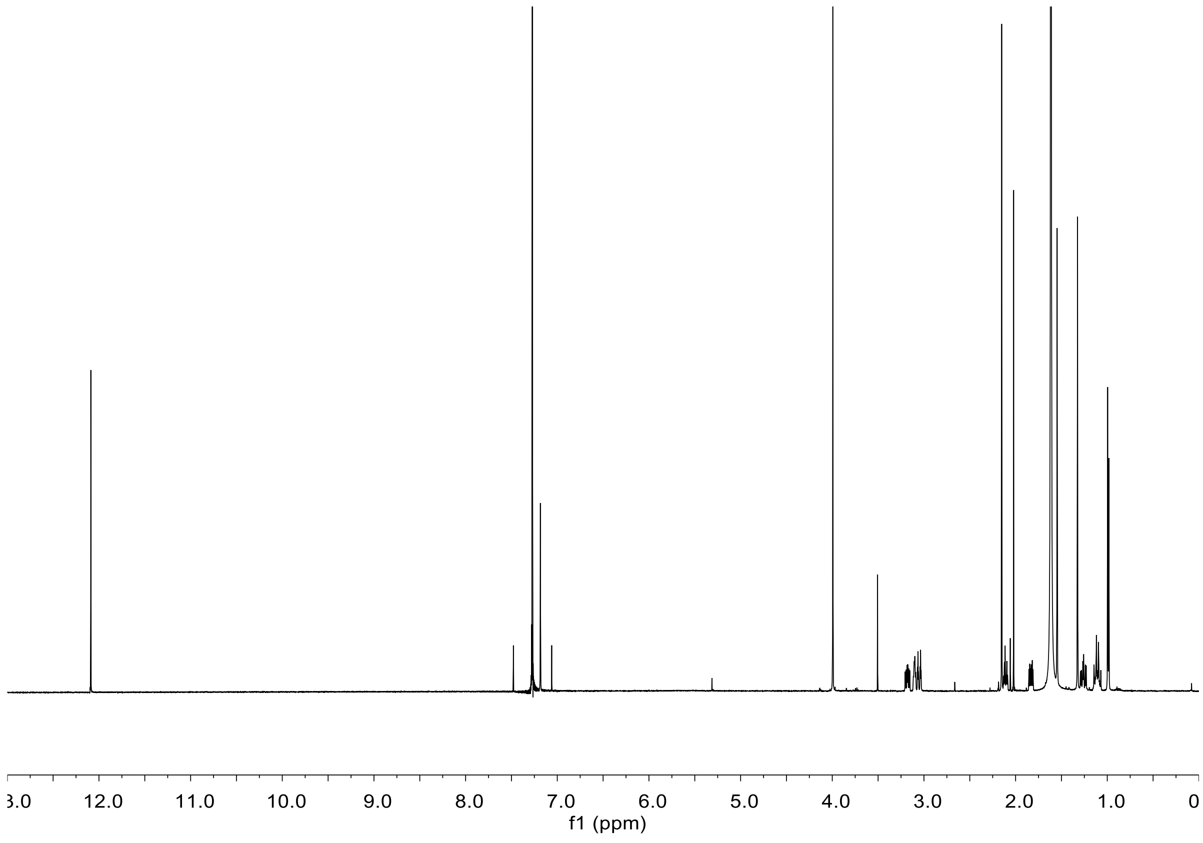

Figure S11. ^{1}H spectrum of ***epi*-1b** in pyridine-d_5

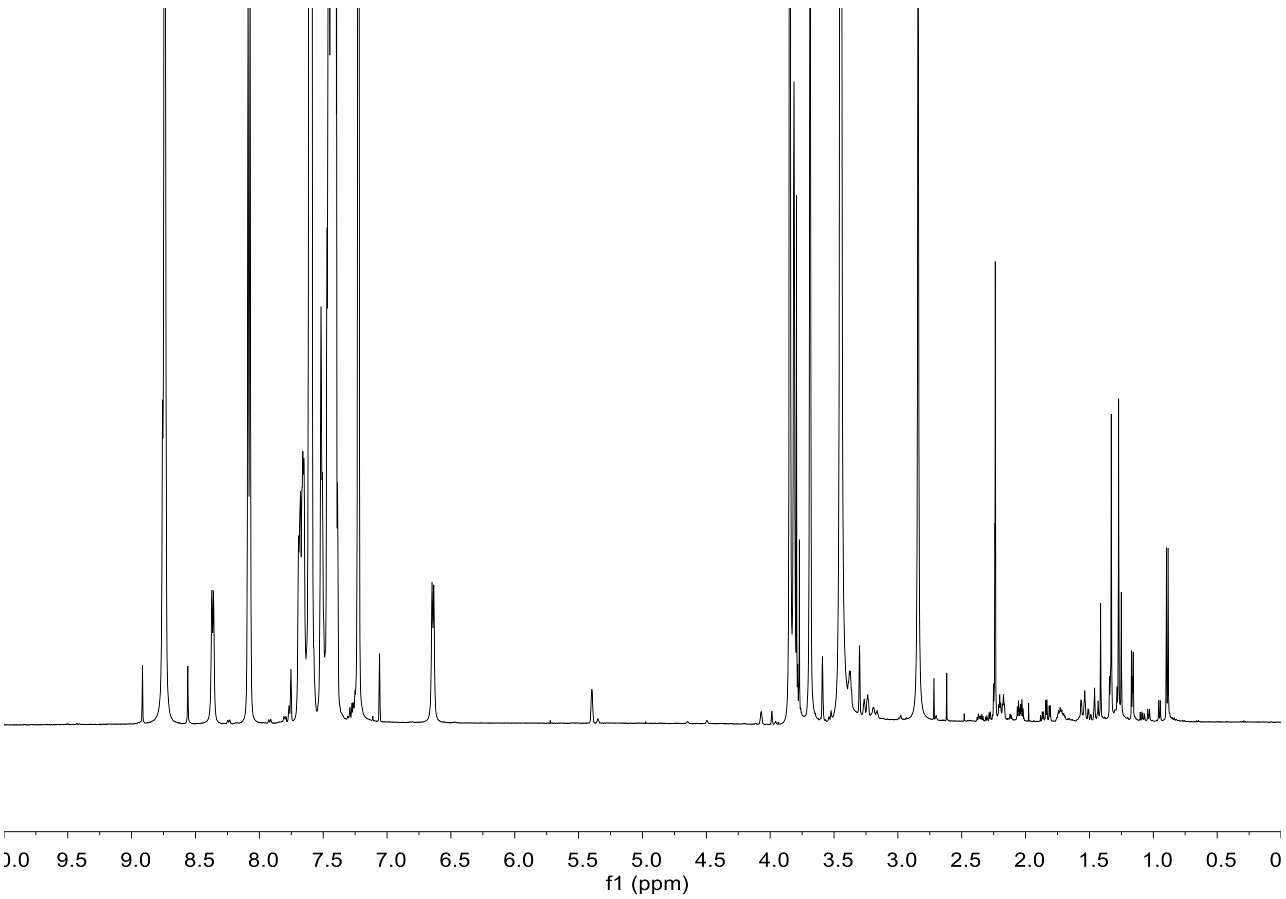

Figure S12. ^{1}H spectrum of ***epi*-1c** in pyridine-d_5

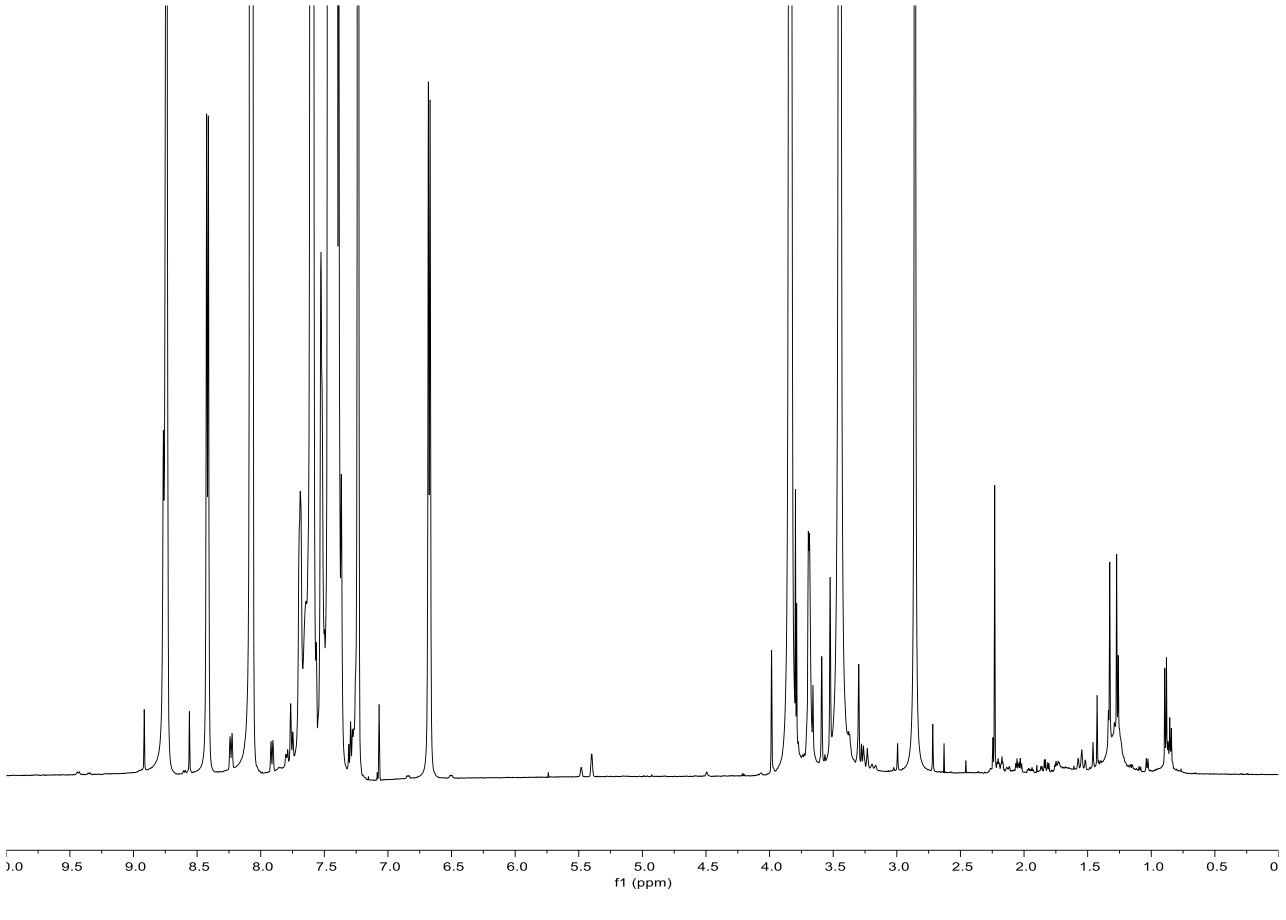

Figure S13. On-line-ABTS^{+} assay of Trolox (a), **1** (b) and **2** (c). Ten milliliters were analyzed by HPLC with a DAD at 210 nm and 268 nm prior to reaction with the ABTS^{+} radical and the analysis of antioxidant potential at 734 nm.

(a)

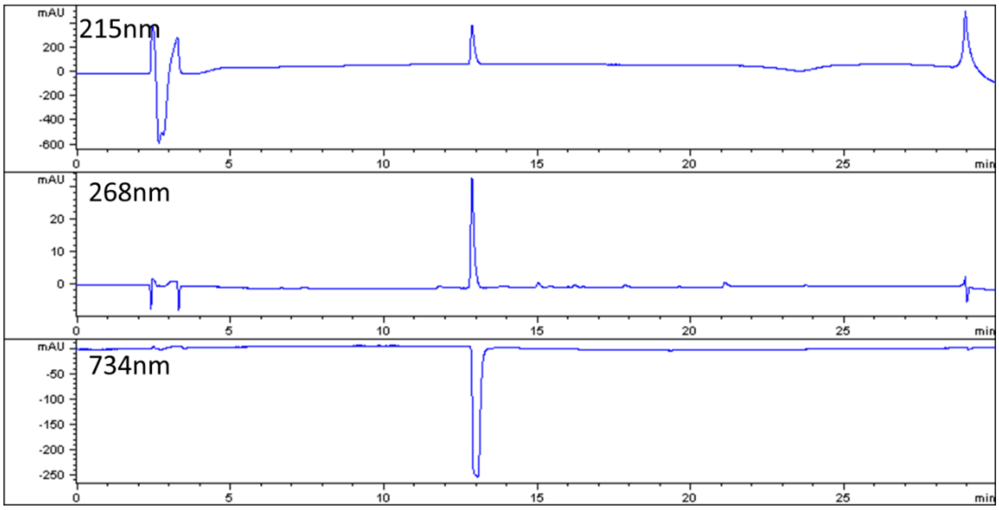

(b)

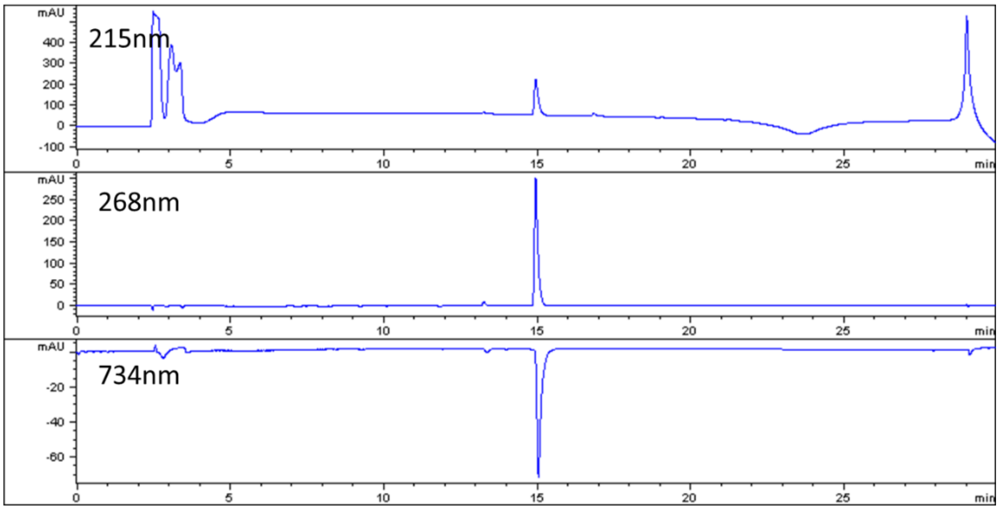

(c)

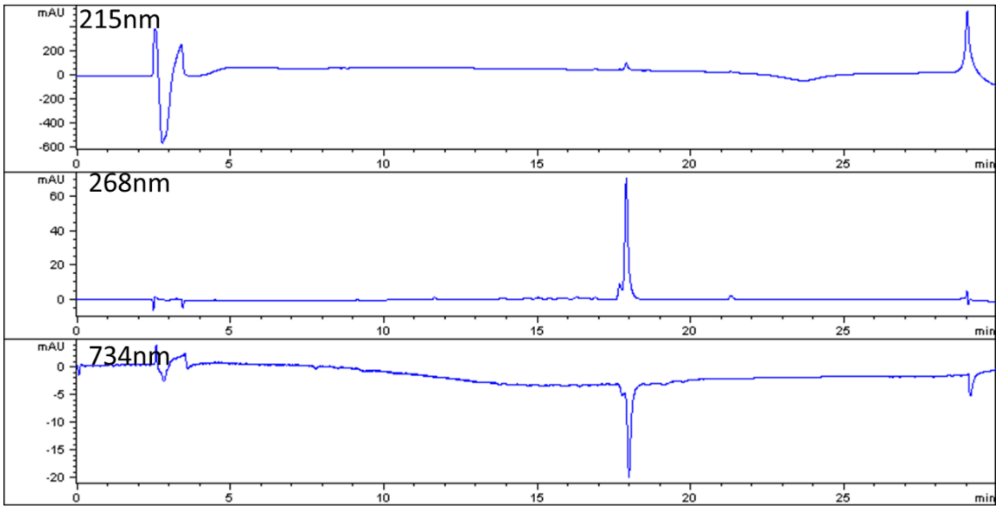

Supplement: Supplementary file 1 [file marinedrugs-16-00090-s001.pdf]
